# Editorial: Innovations in allergy diagnostics and management: a global perspective

**DOI:** 10.3389/falgy.2026.1909853

**Published:** 2026-07-13

**Authors:** Andrijana Nesic, Ivana Prodić, Maja Krstić Ristivojević, Davor Plavec

**Affiliations:** 1 Chair of Molecular Medicine, Institute for Translational Medicine (ITM), Medical School Hamburg (MSH), Hamburg, Germany; 2 Protein Engineering and Biochemistry Department, Institute of Virology, Vaccines and Sera “Torlak”, Belgrade, Serbia; 3 Department of Biochemistry, Faculty of Chemistry, University of Belgrade, Belgrade, Serbia; 4 Medical Faculty, JJ Strossmayer University of Osijek, Osijek, Croatia; 5 PRIMA NOVA, Healthcare Institution, Zagreb, Croatia

**Keywords:** allergic diseases, allergy diagnostics, environmental exposures, *in vitro* diagnostics, molecular allergology, multi-omics, non-invasive biomarkers, personalized medicine

Allergic diseases, including asthma, allergic rhinitis, food allergy, and drug hypersensitivity, represent heterogeneous immune-mediated conditions with a substantial clinical and public health burden. Their diagnosis and management remain challenging because clinical symptoms often overlap, sensitization does not always correspond to clinically relevant allergy, and disease expression is determined by complex interactions among genetic predisposition, epithelial barrier function, immune regulation, environmental exposures, infections, nutritional status, and therapeutic history. These challenges have driven demand for diagnostic and management strategies that are more precise, mechanistically grounded, and globally applicable.

**Figure 1:**
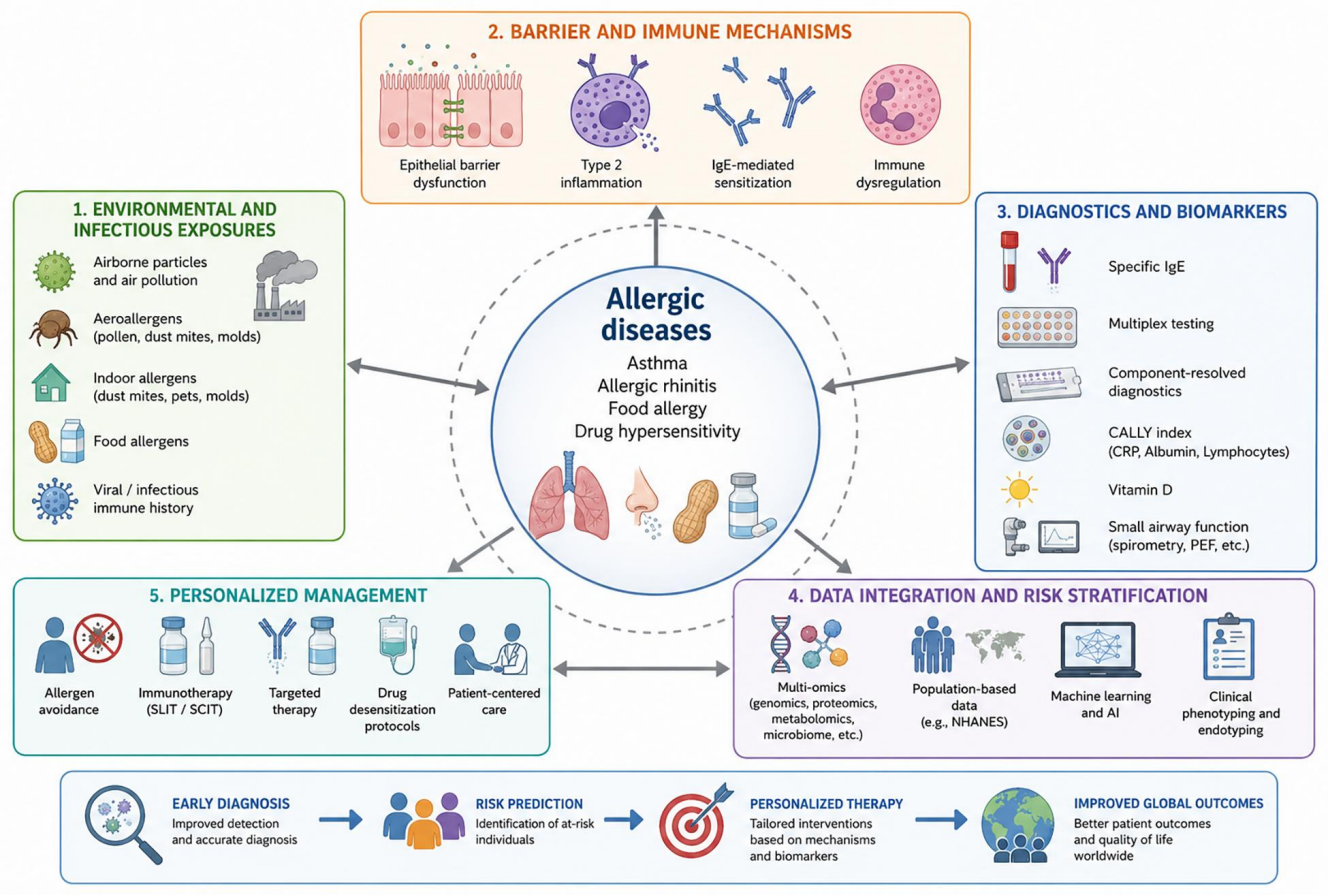
Integrated framework for biomarker-informed diagnosis and personalized management of allergic diseases. The figure summarizes the conceptual framework of the Research Topic, linking environmental and infectious exposures, epithelial barrier dysfunction, immune mechanisms, *in vitro* diagnostics, non-invasive biomarkers, multi-omics and population-level data with personalized management strategies. This integrated approach supports early diagnosis, risk prediction, individualized therapy, and improved patient-centered outcomes across diverse clinical and geographic settings.

The Research Topic “Innovations in Allergy Diagnostics and Management: A Global Perspective” integrates advances in non-invasive biomarkers, *in vitro* diagnostics, environmental assessment, translational models, and personalized therapeutic approaches. Collectively, the contributions reflect a broader transition in allergology-from symptom-based classification toward biomarker-informed, mechanism-driven clinical practice (Figure 1).

A central theme is the refinement of *in vitro* and molecular diagnostic approaches. Hahn et al. compared the RIDA qLine Allergy multiparameter immunoblot with ImmunoCAP specific IgE testing for the identification of clinically relevant food and aeroallergen allergies. Their work emphasizes both the potential value of multiparameter testing and the continuing need for careful clinical interpretation, particularly when broad sensitization profiles, extract-based testing, and cross-reactivity are involved. Complementing this diagnostic perspective, Han et al. reviewed the evolution from conventional skin testing to molecular diagnostics in dust mite allergy. This article highlights how component-resolved diagnostics can improve diagnostic precision, while also underscoring the translational challenges related to assay standardization, clinical relevance, and therapeutic decision-making.

Mechanistic insight into allergic airway disease was provided by Bao et al., who characterized the dual regulatory role of the respiratory epithelium. Rather than a passive physical barrier, the epithelium functions as an active immunological interface integrating allergen signals, environmental pollutants, microbial stimuli, and inflammatory mediators. Barrier dysfunction facilitates sensitization, amplifies type 2 inflammation, and contributes to chronic airway disease. This conceptual framework is directly relevant to biomarker development, since epithelial-derived mediators and measures of barrier integrity may become important tools for early diagnosis, monitoring, and therapeutic targeting.

Asthma heterogeneity was addressed from complementary perspectives. Zhang et al. examined how multi-omics integration, encompassing genomics, transcriptomics, proteomics, metabolomics and microbiome profiling, can delineate asthma phenotypes and endotypes to support individualized treatment. Yang et al. analyzed associations between vaccination history, viral antibody profiles, and asthma prevalence using NHANES data, illustrating the contribution of immune history and epidemiological datasets to asthma risk assessment.

Additional biomarker-oriented studies further support the need for multidimensional asthma evaluation. Liu and Wei investigated the association between the CALLY index, vitamin D, and asthma using NHANES data. Their findings suggest that composite indices reflecting systemic inflammation, immune status, and nutritional balance may have value in asthma-related risk stratification, although prospective validation remains necessary. Hou et al. examined the chronological age at which small airway function begins to decline in a healthy Chinese population. Although performed in a non-asthmatic population, this study is relevant to early respiratory assessment because small airway dysfunction may represent an underrecognized functional marker of future airway vulnerability.

The Research Topic also extends to personalized therapeutic management. Torres Górriz et al. compared rapid and same-day desensitization protocols for hypersensitivity reactions to platinum- and taxane-based chemotherapy, demonstrating that structured risk stratification and individualized desensitization can preserve access to essential oncological therapy without compromising safety.

Taken together, these contributions illustrate that advancing allergy diagnostics requires integration across molecular allergology, epithelial biology, systemic biomarkers, lung function, environmental and infectious exposures, population-based data, and personalized therapeutic protocols. Future research should prioritize prospective validation of candidate biomarkers, harmonization of diagnostic platforms, external validation of computational models, and assessment of clinical utility across diverse geographic and socioeconomic settings. Such efforts are essential to ensure that innovations in allergy diagnostics are not confined to specialized centers but become applicable to broader patient populations.

In conclusion, this Research Topic highlights the ongoing transformation of allergology into a more precise, mechanism-informed, and patient-centered discipline. By connecting diagnostic innovation with biomarker discovery, environmental context, translational insight, and individualized therapy, the included studies contribute to a more integrated framework for the early diagnosis and management of allergic diseases worldwide.

